# Massive Integration of Planktonic Cells within a Developing Biofilm

**DOI:** 10.3390/microorganisms9020298

**Published:** 2021-02-02

**Authors:** Nay El-Khoury, Imene Bennaceur, Emilie Verplaetse, Stéphane Aymerich, Didier Lereclus, Mireille Kallassy, Michel Gohar

**Affiliations:** 1 Micalis Institute, Université Paris-Saclay, INRAE, AgroParisTech, 78350 Jouy-en-Josas, France; Nay.El-Khoury@inrae.fr (N.E.-K.); imene.bennaceur@hotmail.fr (I.B.); Emilie.Verplaetse@inrae.fr (E.V.); stephane.aymerich@inrae.fr (S.A.); didier.lereclus@inrae.fr (D.L.); 2 Biotechnology Laboratory, UR EGP, Faculty of Science, Saint Joseph University, B.P. 11-514 6 Riad El Solh, Beirut 1107 2050, Lebanon; mireille.kallassy@usj.edu.lb

**Keywords:** biofilm, *Bacillus cereus*, *Bacillus thuringiensis*, recruitment, heterogeneity

## Abstract

During biofilm growth, the coexistence of planktonic and sessile cells can lead to dynamic exchanges between the two populations. We have monitored the fate of these populations in glass tube assays, where the *Bacillus thuringiensis* 407 strain produces a floating pellicle. Time-lapse spectrophotometric measurement methods revealed that the planktonic population grew until the pellicle started to be produced. Thereafter, the planktonic population decreased rapidly down to a value close to zero while the biofilm was in continuous growth, showing no dispersal until 120 h of culture. We found that this decrease was induced by the presence of the pellicle, but did not occur when oxygen availability was limited, suggesting that it was independent of cell death or cell sedimentation and that the entire planktonic population has integrated the biofilm. To follow the distribution of recruited planktonic cells within the pellicle, we tagged planktonic cells with GFP and sessile cells with mCherry. Fluorescence binocular microscopy observations revealed that planktonic cells, injected through a 24-h-aged pellicle, were found only in specific areas of the biofilm, where the density of sessile cells was low, showing that spatial heterogeneity can occur between recruited cells and sessile cells in a monospecies biofilm.

## 1. Introduction

Biofilm growth can be the result of sessile cell division or of the integration of incoming bacteria (planktonic cell recruitment). Planktonic cell recruitment is extensively described in some multispecies biofilms, such as the dental plaque. In this oral biofilm, the sequential recruitment of secondary or tertiary colonizers occurs through specific interactions with pioneer species [1]. For instance, *Porphyromonas gingivalis* fimbriae interact with a glyceraldehyde 3-phosphate deshydrogenase produced by *Streptococcus oralis*, a primary colonizer of the tooth enamel [2]. Planktonic cell integration within an existing biofilm enables bacteria devoid of biofilm-forming capacities, including pathogens, to colonize and sustain their persistence in numerous environments. Cell immigration into a mature biofilm has also a significant impact on microbial ecology since it promotes expansion of the genetic pool in the resident biofilm by horizontal DNA transfer between species [1,3,4]. It also enhances the resilience to adverse environmental conditions by increasing the variety of metabolic processes within the community and can lead to spatial heterogeneity of the biofilm through species stratification, according to their metabolic requirements [5].

*Bacillus cereus* and *Bacillus thuringiensis* are motile, facultative aerobic, spore-forming bacteria that belong to the *Bacillus cereus sensu lato* group [6]. Both species are genetically very close, but while *B. thuringiensis* is used as a natural pesticide, having a wide range of entomopathogenic activity due to parasporal crystal proteins [7], *B. cereus* is a human pathogen, involved in food poisoning [8] and systemic or local infections [9]. *B. thuringiensis* and *B. cereus* are able to produce dense biofilms at the air–liquid interface [10]. The air–liquid interface is a suitable environment for the development of aerobic microorganisms, since it provides access to oxygen. In *Shewanella oneidensis* or *Pseudomonas fluorescens*, oxygen is required for pellicle formation at the air-liquid interface [11,12]. Planktonic bacteria movement toward the biofilm requires motility. *Bacillus subtilis* immotile mutant strain was shown to have a delay in pellicle formation compared to the wild-type strain [13], and immotile mutants of *B. thuringiensis* strain 407 do not produce biofilms [10].

Although the recruitment of planktonic cells by the biofilm has been the subject of a number of reports, dynamic studies of the interaction between the two populations when a pellicle is produced at an air–liquid interface have been seldom conducted. The objective of this study was to determine the fate of a planktonic population during the development of the biofilm at the air–liquid interface, in *B. thuringiensis*.

## 2. Materials and Methods

### 2.1. Strains and DNA Manipulation

Strains used in this study are listed in Table S1. The *B. thuringiensis* 407 Cry^−^ strain (*Bt407*) has been cured of its Cry plasmid [14]. *Bt407* forms thick biofilms and is genetically similar to *B. cereus* strains [15]. The *Bt407* ∆Spo0A strain was constructed by insertion of a kanamycin resistance cassette in spo0A [16]. The *B. cereus* strains included in this work were ATCC14579, AH829 and ATCC10987 [17].

Two strains were used for epifluorescence microscopy observations. The GFP-tagged *Bt407* strain, expressing constitutively GFP, was constructed by inserting *gfp* in the alpha amylase gene BTB_c12100 of the *Bt407* strain (chromosome accession number CP003889). DNA fragments corresponding to the chromosomal DNA regions upstream and downstream of BTB_c12100 were generated by PCR using the primer pairs AmyAFW–AmyARV and AmyBFW–AmyBRV, respectively (Supplementary Materials Table S1). The *gfp* coding sequence, including the SarA promoter sequence, was amplified from pCM11 [18] using primers sGFPFW and sGFPRV (Table S2). The amplified DNA fragments and the *sarA–gfp* fragment were cloned between the *HindIII* and the *BamHI* sites of plasmid pRN5101 [19]. The resulting plasmid was used to transform the *Bt407* wild-type strain by electroporation [14], and *sarA-gfp* was inserted into the alpha amylase gene after allelic exchange by homologous recombination [20]. The corresponding strain was designated strain *Bt407-gfp*. The mCherry-tagged *Bt407* strain was constructed as follows. A 506-bp *SphI-XbaI* DNA fragment containing the *aphaIII* promoter was amplified from the pDG783 plasmid [21] by PCR, using the primers pAphaIII-F and pAphaIII-R (Table S2), and inserted in the pHT1618 plasmid [22]. A *KpnI-EcoRI* DNA fragment including an optimized RBS sequence and the *mCherryLGC* gene was generated by PCR from pHT304-18 mCherry [23] with the mCherry-F and the mCherry-R primers and inserted in pHT1618-pAphaIII. From the resulting plasmid, a 1268-bp *SphI-EcoRI* DNA fragment was amplified by PCR and inserted in pAT28 [24], giving rise to the pAT28-mCherry plasmid. This plasmid was transformed in the *Bt407* strain to obtain the *Bt407-mCherry* strain.

### 2.2. Time-Course of Planktonic and Biofilm Population Growth

A 100-mL Erlenmayer culture flask, filled with 10 mL LB, was seeded with one colony and grown at 30 °C, 175 rpm until the exponential growth phase was reached. These pre-cultures were diluted to an OD_600_ of 0.01 into fresh HCT culture medium [25] and 2 mL of this dilution was distributed in UV-sterilized 3.5-mL glass cuvettes (1 × 1 mm section) sealed with a sterile cotton plug. The OD_600_ of the planktonic population below the biofilm was recorded in situ in time-lapse using a Shimadzu UV2501 spectrophotometer, for a duration of 48 h at 2-min intervals, and at a regulated temperature of 30 °C. In one experiment, 250 µL of sterile mineral oil (paraffin oil) was poured on top of the culture medium. Each experiment was repeated three times, and representative curves are shown. Biofilms were produced in glass tubes as described earlier [10]. Briefly, pre-cultures, obtained as specified above, were diluted to an OD_600_ of 0.01 into HCT. Two milliliters of the diluted culture was distributed in UV-sterilized glass tubes (10.8 mm internal diameter, 64 mm height). After different times of incubation (from 8 to 60 h) at 30 °C, the 2 mL culture medium was removed using a Pasteur pipette and the OD_600_ of the floating biofilm, thoroughly resuspended and vortexed in 2 mL PBS, was measured. The biofilm OD_600_ is linearly and significantly related to the number of vegetative cells or spores contained within it, the two latter having the same absorbance [26]. Representative results of three replications are shown. In the two devices (biofilm and planktonic growth measurement), the same culture medium, incubation temperature and volume were used. The cross-section surfaces were similar: 0.92 cm^2^ vs. 1 cm^2^. The only difference was that the cross-section was a square for planktonic culture growth measurement and a circle for biofilm growth measurement, which makes comparisons between the two sets of results possible.

### 2.3. Recruitment of Gfp-Tagged Planktonic Cells in a mCherry-Tagged Biofilm

Biofilms were produced in the same way as above, except that the glass tube height was 30 mm instead of 64 mm (same diameter). The m-Cherry labeled *Bt407* strain was used to form the biofilm, and 50 μL of a gfp-labelled *Bt407* planktonic cell culture was injected in the 2 mL culture medium through the 12-h-old pellicle and near the bottom of the tube, using a 26-needle gauge and a micro-manipulator to avoid pellicle disturbance. The injected planktonic cells were taken from an exponential growth phase culture in HCT medium at an OD_600_ of 1.0. Two mL of this culture was centrifuged at 3000 rpm and resuspended in 0.1 mL of sterile water (final OD_600_ after injection: 0.5). The glass tubes were observed 24 h later with a fluorescence stereomicroscope.

### 2.4. Time-Course of the Pellicle Formation

A *Bt407 wt* culture in exponential phase was diluted to an OD_600_ of 0.01 into HCT medium. Then, 2.5 mL of the diluted culture was distributed in a sterile 5-mL beaker (1.8 cm diameter) closed by a sterile glass slide and incubated at 30 °C. Pictures were taken every 10 min with a digital camera for a duration of 48 h. Pictures were processed by Adobe Photoshop^®^ CS6 to build an mp4 video file showing the pellicle growth over time.

## 3. Results

### 3.1. The Planktonic Population Decreases When the Biofilm Grows

**Figure 1:**
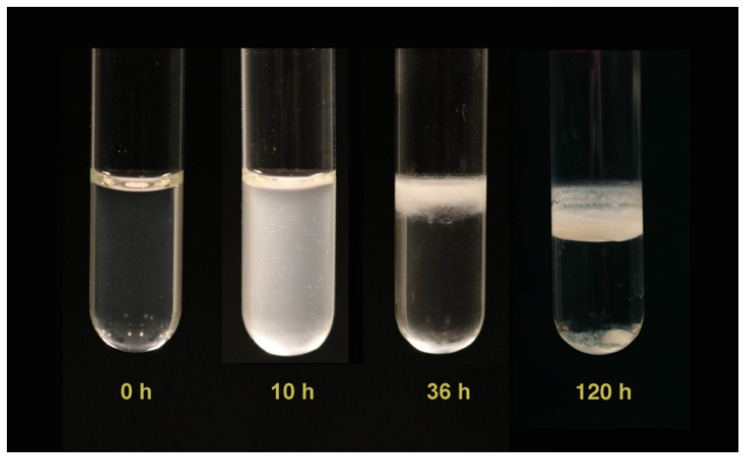
Biofilm formation and planktonic growth in glass tubes. The *Bt407* strain was grown, in static conditions at 30 °C, in HCT medium and in glass tubes. Pictures were taken at the start of the experiment (0 h), and after 10, 36 or 120 h of incubation.

The *Bt407 wt* strain, when cultured in static conditions, produces a floating pellicle that covers the whole liquid surface [27]. In the glass tube assays, no pellicle was formed after 10 h of culture but the culture medium was turbid, consecutively to the planktonic population growth (Figure 1). However, after 36 h or 120 h of incubation, a dense pellicle was produced and the culture medium reversed to a transparent state similar to the one observed at the onset of the experiment (0 h).

**Figure 2:**
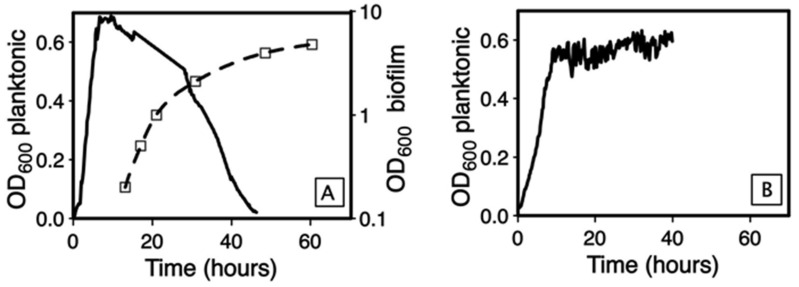
Time-course of the OD_600_ for planktonic or sessile populations in glass tube assay. (**A**): Strain *Bt407*; (**B**): *Bt407*∆Spo0A. Plain line: planktonic population; dotted line: sessile population. Left *y*-axis: planktonic population OD_600_, linear scale; Right *y*-axis: biofilm OD_600_, log-scale.

This observation suggested that the planktonic population vanished while the biofilm grew. To follow the fate of the planktonic population during biofilm growth, we recorded, by spectrophotometry, the time-course of bacterial density both in the pellicle and in the planktonic population (Figure 2A). In the first hours of culture, the planktonic biomass increased rapidly until it reached a plateau at OD_600_ 0.7, between 6.5 and 12 h of incubation. At the end of this plateau, the biofilm could be detected for the first time and started its exponential growth, while the planktonic population density began to decrease. At the end of the biofilm growth, after 48 h of incubation, the planktonic population density was nearly down to zero (OD_600_ 0.02). To confirm that the onset of the planktonic population decrease is simultaneous to biofilm growth, we monitored in the *Bt407* strain the pellicle formation for 41 h, in time-lapse and under a stereomicroscope. Pictures were taken every 10 min and assembled within a video (Video S1, Supplementary Materials). The movie shows that the pellicle could be seen at the liquid surface between 14 and 15 h after the start of the culture, and that after 16 h of incubation, the biofilm growth increases exponentially, forming thick protrusions. After 30 h of incubation, the biofilm was fully completed. An analysis of the movie images shows that the surface covered by the biofilm increases sharply between 14 and 17 h, after which the whole surface is covered (Figure S1). The subsequent development of the biofilm can therefore be attributed an increase in thickness.

### 3.2. The Biofilm Is the Cause of the Planktonic Population Decrease

**Figure 3:**
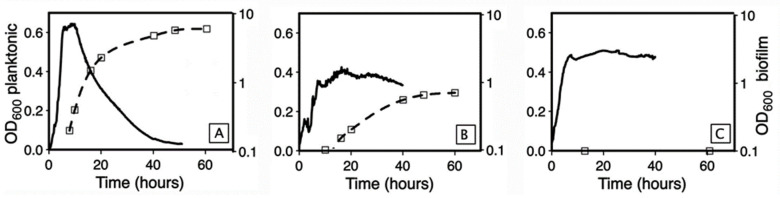
Steepness of the planktonic population decrease as a function of biofilm formation ability. (**A**): Strain ATT10987; (**B**): strain 1TCC14579; (**C**): strain AH829. Plain line: planktonic population; dotted line: sessile population. **Left** *y*-axis: planktonic population OD_600_, linear scale; **Right** *y*-axis: biofilm OD_600_, log-scale. Assays in glass tubes.

Because the start of planktonic population decrease was coincident with the onset of biofilm growth, we hypothesized that the planktonic population decrease was related to biofilm formation. To test this hypothesis, we used the sporulation-deficient mutant *Bt407* ∆Spo0A, which is unable to form a biofilm [28]. As shown in Figure 2B, the planktonic population grew rapidly until it reached a plateau at OD_600_ 0.6, and thereafter showed no decrease until 48 h of incubation. Therefore, the decrease in the planktonic population is a consequence of biofilm formation. To determine if the speed of decrease depends on the biofilm biomass, we repeated the experiment with a set of *B. cereus* strains with variable abilities to form a pellicle (Figure 3).

**Figure 4:**
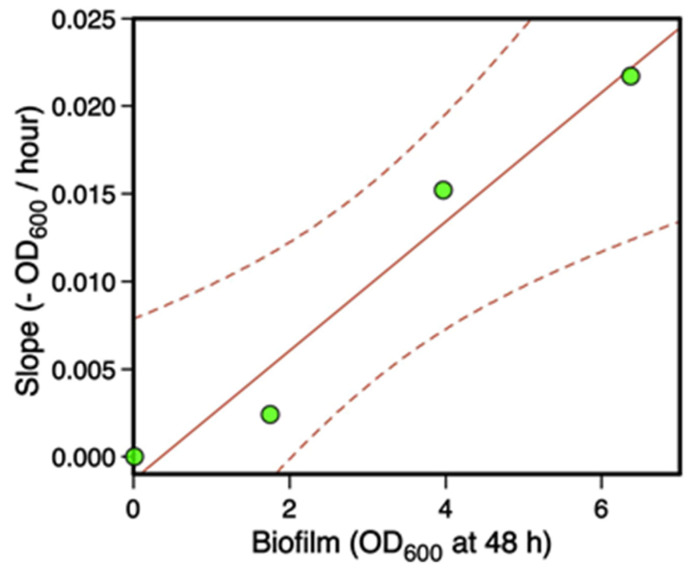
Relation between the slope (OD_600_/h) of the planktonic population decrease and the biomass of 48-h-aged biofilms. Data were obtained from strains *Bt407,* ATCC10987, ATCC14579 and AH829. The linear regression (slope vs. biofilm) was significant (*p* < 0.05, r = 0.98).

The slope of planktonic OD_600_ decrease was steep for strain ATCC10987, which produces dense biofilms [29], intermediate for strain ATCC14579, which produces poor biofilms, and null for strain AH829, which is unable to form a biofilm. The relationship between the slope of planktonic OD_600_ decrease and the ability to form a biofilm was linear (r = 0.98, *p* < 0.05) (Figure 4).

### 3.3. The Planktonic Population Is Recruited within the Biofilm

**Figure 5:**
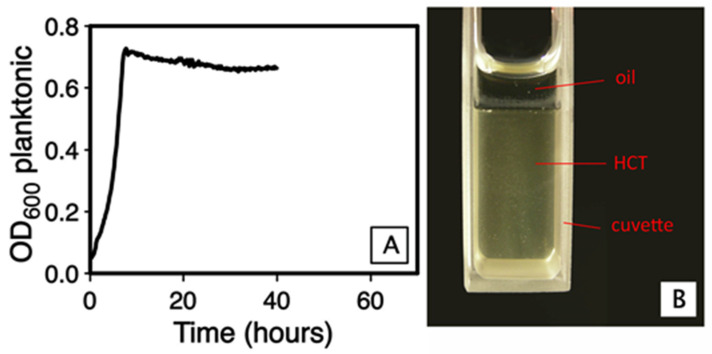
Growth of *Bt407* in micro-aerobic condition. The culture was performed in static condition at 30°, in HCT medium and in a glass cuvette. (**A**): Time-course of the OD_600_ of the planktonic population. (**B**): Picture of the cuvette after 48 h of incubation.

Planktonic population decrease might be attributed to cell lysis, cell sedimentation or cell integration within the growing biofilm (recruitment). We measured the optical density of cells sedimented at the bottom of the tube. When resuspended in the initial culture volume, the sediment had an OD_600_ of 0.16 ± 0.02. It should be noted that this sediment includes not only cells from the planktonic population but also biofilm pieces detached from the floating pellicle, which makes sedimentation unlikely to explain, by itself, the drop in planktonic OD_600_. Cell mortality was difficult to estimate through measurement of released DNA or stable cytosolic proteins, because *B. thuringiensis* secretes a high number of degradative enzymes, including proteases and nucleotidases [30]. However, the reason that the biofilm could induce planktonic bacterial mortality is likely to be the limitation of oxygen exchanges, consecutively to the presence of a pellicle covering the whole surface since, even in the absence of a pellicle, the consumption of oxygen by the planktonic population leads to a low oxygen concentration in the culture medium (Figure S2). To test this hypothesis, we followed over time the OD_600_ of the planktonic population in a glass cuvette, in which the culture medium was covered by a layer of mineral oil, to suppress oxygen exchange. In this condition, the planktonic population grew until it reached a peak at OD_600_ 0.7, but did not decrease afterward (Figure 5A). After 48 h of incubation, no biofilm was produced and the culture medium was turbid, showing the presence of a planktonic population (Figure 5B). Therefore, the micro-aerobiotic condition which prevails beneath the biofilm does not induce bacterial mortality, which means that the planktonic population decrease observed during biofilm growth is a consequence of recruitment.

### 3.4. Recruited Bacteria Are Located in Specific Areas of the Biofilm

**Figure 6:**
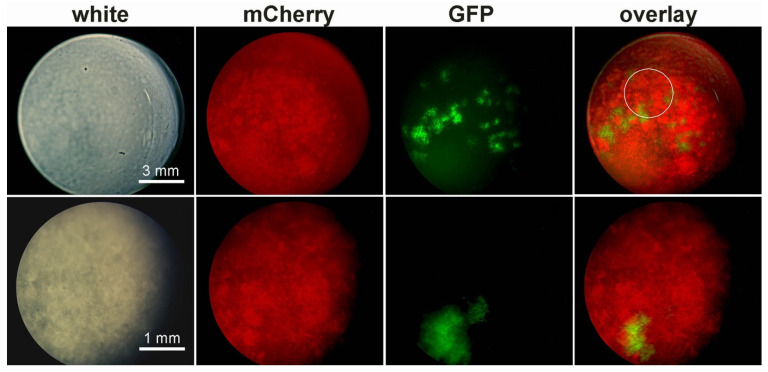
Recruitment of a GFP-tagged planktonic population in an mCherry-tagged biofilm. The biofilm, formed at the air–liquid interface, was observed with a fluorescence binocular microscope. Upper row, view of whole biofilm surface. Bottom row, 3-fold magnification of a region near the center of the biofilm, depicted by the white circle in the overlay of the upper row. The white bar gives the scale. White: white light; Overlay: overlay of the mCherry and the GFP channels.

To observe the integration of planktonic bacteria within the biofilm, we injected a GFP-tagged planktonic population through a 12-h-aged mCherry-tagged biofilm pellicle, in the culture medium beneath the biofilm. The biofilm was observed 24 h later with a fluorescence binocular microscope. The pellicle structure as seen in white light was heterogeneous, with clusters of high biomaterial density surrounded by material of lower density (Figure 6). Live cells in the resident biofilm appeared as red fluorescent macrocolonies scattered throughout the whole biofilm, in areas of high biomaterial density. Interestingly, green fluorescent macrocolonies were found in specific areas, where the density of biomaterial was high, but the density of red fluorescent cells was low (Figure 6, lower row).

## 4. Discussion

During the formation of a biofilm in a closed environment, two populations, sessile and planktonic, coexist and can exchange. Cells from the biofilm can migrate to the planktonic population while cells from the planktonic population can enter the biofilm. We found that, in the *B. thuringiensis* strain *Bt407*, which produces a floating pellicle, the planktonic population density drops to nearly zero when the biofilm reaches a steady state of growth. We used spectrophotometric methods to follow, in separate experiments but in very similar devices, the time-course of both populations. The planktonic population decrease starts when the biofilm initiates its growth. This observation is supported by time-lapse photography of the pellicle formation, which reveals that the pellicle enters its exponential growth phase at the time when the planktonic population starts its decrease. However, the planktonic growth curve has already reached a maximum before the biofilm growth started, which suggests that planktonic bacteria were already in the stationary phase of growth because of a deficiency in nutrients or in oxygen. However, since the planktonic population decrease did not occur in the Spo0A mutant, unable to produce a biofilm but motile [28], it should be a consequence of the pellicle formation. Using a set of strains with variable abilities to form a biofilm, we found that the rate of the planktonic population decrease was significantly correlated to the amount of biofilm produced, which confirms further that the biofilm is the cause of the planktonic population decrease.

The pellicle, which contains a high density of cells, is likely to reduce dramatically the dissolved oxygen in the culture medium, a situation which could lead to cell sedimentation or cell death. In *P. fluorescens*, pellicle colonizers consume oxygen, resulting in anoxia at a depth below 1.2 mm after a few hours [31]. However, *B. cereus*, which is closely related to *B. thuringiensis*, is a facultative aerobic bacterium shown to survive, although with a very low growth rate, in anaerobic conditions [32]. To determine if low oxygen availability could, in *B. thuringiensis*, explain the planktonic population decrease, we limited air–liquid exchange with a layer of mineral oil applied on the culture medium surface, a method already used in *P. fluorescens* to show that oxygen availability is a limiting factor for pellicle formation [12]. Despite oxygen limitation, the planktonic population did not decrease, at least until 48 h of culture, showing that the decrease observed when a pellicle is formed is independent of biofilm-induced changes in oxygen concentration. Therefore, the most likely explanation for the planktonic population decrease is its recruitment in the biofilm. However, recruitment is only part of the biofilm growth in *B. thuringiensis*, since the biofilm continued its growth while the planktonic population was already very low. In contrast, in some species such as *Legionella pneumophila*, the biofilm development relies mainly on continuous recruitment rather than on sessile cell division [33].

**Figure 7:**
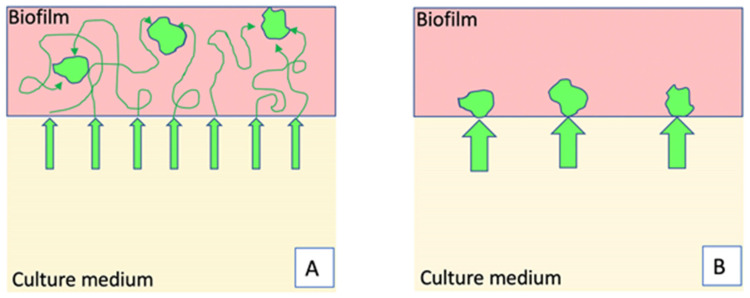
Hypotheses to explain the integration of planktonic cells in specific areas of the biofilm. (**A**): Planktonic cells enter deeply in the pellicle, are motile, and settle in suitable areas. (**B**): Planktonic cells enter the pellicle directly in specific spots, located at the biofilm lower side.

We found that, in a young biofilm and while the planktonic population is still high, immigrant cells were located in specific areas of the biofilm, where the density of sessile cells was low. In *Listeria monocytogenes*, the biofilm exopolysaccharide matrix has been reported to prevent the immigration of planktonic cells [34], which could lead, in *B. thuringiensis*, to the confinement of incoming cells in areas of low biomaterial density. Alternatively, the heterogeneous distribution of immigrant cells might be due to the presence in the pellicle of areas in which nutrients and oxygen are present in higher quantities. Aerotaxis and chemotaxis could attract, in these micro-environments, cells from elsewhere in the pellicle or from the culture medium beneath the pellicle. Since we observed the pellicle with a fluorescence stereomicroscope, because confocal microscopy could not be used on a live floating pellicle, we did not determine the position of immigrant cells on the Z-axis. Therefore, planktonic cells could have migrated in the whole pellicle and reached secondarily suitable areas (Figure 7A). Because the fluorescence microscopy observations took place 12 h after the injection of planktonic cells in the culture medium beneath the pellicle, the motile cells had enough time to join suitable areas of the pellicle and turn into sessile cells. Alternatively, planktonic cells could have migrated directly to suitable areas located on the pellicle basal side (Figure 7B). Immigrant cells were indeed shown to remain motile within the biofilm, at least for a few hours in *B. thuringiensis* [35], and were reported to migrate to the pellicle in an oxygen gradient-dependent way in *B. subtilis* [13]. Interactions of freshly recruited bacteria with the biofilm matrix, as described in *Vibrio cholerae* [36,37], are unlikely to occur in the *Bt407* biofilm, since these interactions would limit bacterial movements.

While planktonic cells migrated to the surface to form a pellicle, there was no bacterial dispersal from the *Bt407* biofilm, at least until 120 h of culture (Figure 1). In addition, no rise in the planktonic population was observed, even after 48 h of culture. Cell dispersal from the biofilm in *B. cereus* was shown to be strain-dependent. The ATCC14579 and the ATCC10789 strains were both capable of forming pellicles in Y1 medium, but only the ATCC14579 biofilm dispersed after 48 h of culture [38]. Bacterial dispersal from the biofilm in *B. subtilis* usually occurs during biofilm maturation, when cells “escape” the biofilm because nutrient availability becomes scarce [39]. The *Bt407* biofilm appears to be quite resilient to nutrient deprivation and can survive for at least 96 h of culture in the same conditions as those used here [26]. One possible explanation for this high resilience is that in this strain, the biofilm, after 48 h of culture, contains mainly matrix components and spores [26], and could therefore be a long-lasting structure in which the biofilm protective properties enhance spores’ resistance to antimicrobials and to adverse environmental conditions.

## 5. Conclusions

In conclusion, we have shown in this work that, during the formation of a biofilm in static conditions at the air–liquid interface, one-way exchanges occur between the planktonic population and the biofilm. In the *Bt407* strain, the whole planktonic population is integrated in the biofilm while no biofilm dispersal is observed. Once integrated, the planktonic population does not mix with sessile cells but is located in specific clusters of the biofilm pellicle. These results provide a useful example of the massive immigration of planktonic cells in specific areas of the biofilm, therefore contributing to its spatial heterogeneity.

## Data Availability

Not applicable.

## Supplementary Materials

The following are available online at https://www.mdpi.com/2076-2607/9/2/298/s1, Table S1: List of strains used in this study, Table S2: List of primers used in this study, Video S1: Time-lapse observation of the *Bt407* wt pellicle formation, Figure S1: Area covered by the biofilm as a function of time, Figure S2: Dissolved oxygen concentration in the culture medium during planktonic growth.

## References

[1] Kolenbrander P.E., Palmer R.J., Periasamy S., Jakubovics N.S. (2010). Oral multispecies biofilm development and the key role of cell-cell distance. Nat. Rev. Microbiol..

[2] Kolenbrander P.E., Palmer R.J., Rickard A.H., Jakubovics N.S., Chalmers N.I., Diaz P.I. (2006). Bacterial interactions and successions during plaque development. Periodontology 2000.

[3] Olsen I., Tribble G.D., Fiehn N.-E., Wang B.-Y. (2013). Bacterial sex in dental plaque. J. Oral Microbiol..

[4] Roberts A.P., Cheah G., Ready D., Pratten J., Wilson M., Mullany P. (2001). Transfer of TN916-like elements in microcosm dental plaques. Antimicrob. Agents Chemother..

[5] Nadell C.D., Xavier J.B., Foster K.R. (2009). The sociobiology of biofilms. Fems Microbiol. Rev..

[6] Ehling-Schulz M., Lereclus D., Koehler T.M. (2019). The *Bacillus cereus* Group: *Bacillus* Species with Pathogenic Potential. Microbiol. Spectr..

[7] Schnepf E., Crickmore N., Van Rie J., Lereclus D., Baum J., Feitelson J., Zeigler D.R., Dean D.H. (1998). *Bacillus thuringiensis* and its pesticidal crystal proteins. Microbiol. Mol. Biol. Rev..

[8] Kotiranta A., Lounatmaa K., Haapasalo M. (2000). Epidemiology and pathogenesis of *Bacillus cereus* infections. Microbes. Infect..

[9] Bottone E.J. (2010). *Bacillus cereus*, a volatile human pathogen. Clin. Microbiol. Rev..

[10] Houry A., Briandet R., Aymerich S., Gohar M. (2010). Involvement of motility and flagella in *Bacillus cereus* biofilm formation. Microbiology.

[11] Armitano J., Mejean V., Jourlin-Castelli C. (2013). Aerotaxis governs floating biofilm formation in *Shewanella Oneidensis*. Environ. Microbiol..

[12] Spiers A.J., Bohannon J., Gehrig S.M., Rainey P.B. (2003). Biofilm formation at the air-liquid interface by the Pseudomonas fluorescens SBW25 wrinkly spreader requires an acetylated form of cellulose. Mol. Microbiol..

[13] Holscher T., Bartels B., Lin Y.C., Gallegos-Monterrosa R., Price-Whelan A., Kolter R., Dietrich L.E.P., Kovacs A.T. (2015). Motility, Chemotaxis and Aerotaxis Contribute to Competitiveness during Bacterial Pellicle Biofilm Development. J. Mol. Biol..

[14] Lereclus D., Arantes O., Chaufaux J., Lecadet M. (1989). Transformation and expression of a cloned delta-endotoxin gene in *Bacillus thuringiensis*. Fems Microbiol. Lett..

[15] Tourasse N.J., Helgason E., Okstad O.A., Hegna I.K., Kolsto A.B. (2006). The *Bacillus cereus* group: Novel aspects of population structure and genome dynamics. J. Appl. Microbiol..

[16] Lereclus D., Agaisse H., Gominet M., Chaufaux J. (1995). Overproduction of encapsulated insecticidal crystal proteins in a *Bacillus thuringiensis spo0A* mutant. Biotechnology.

[17] Helgason E., Tourasse N.J., Meisal R., Caugant D.A., Kolsto A.B. (2004). Multilocus sequence typing scheme for bacteria of the *Bacillus cereus* group. Appl. Environ. Microbiol..

[18] Lauderdale K.J., Malone C.L., Boles B.R., Morcuende J., Horswill A.R. (2010). Biofilm dispersal of community-associated methicillin-resistant *Staphylococcus aureus* on orthopedic implant material. J. Orthop. Res..

[19] Lereclus D., Vallade M., Chaufaux J., Arantes O., Rambaud S. (1992). Expansion of insecticidal host range of *Bacillus thuringiensis* by in vivo genetic recombination. Biotechnology.

[20] Lecadet M.M., Chaufaux J., Ribier J., Lereclus D. (1992). Construction of Novel *Bacillus thuringiensis* Strains with Different Insecticidal Activities by Transduction and Transformation. Appl. Environ. Microbiol..

[21] Guerout-Fleury A.M., Shazand K., Frandsen N., Stragier P. (1995). Antibiotic-resistance cassettes for *Bacillus subtilis*. Gene.

[22] Lereclus D., Arantes O. (1992). spbA locus ensures the segregational stability of pTH1030, a novel type of gram-positive replicon. Mol. Microbiol..

[23] Verplaetse E., Slamti L., Gohar M., Lereclus D. (2015). Cell Differentiation in a *Bacillus thuringiensis* Population during Planktonic Growth, Biofilm Formation, and Host Infection. MBio.

[24] Trieu-Cuot P., Carlier C., Poyart-Salmeron C., Courvalin P. (1990). A pair of mobilizable shuttle vectors conferring resistance to spectinomycin for molecular cloning in *Escherichia coli* and in gram-positive bacteria. Nucleic Acids Res..

[25] Lecadet M.M., Blondel M.O., Ribier J. (1980). Generalized Transduction in *Bacillus thuringiensis* var. berliner using Bacteriophage CP-54Ber. J. Gen. Microbiol..

[26] El-Khoury N., Majed R., Perchat S., Kallassy M., Lereclus D., Gohar M. (2016). Spatio-Temporal Evolution of Sporulation in *Bacillus thuringiensis* Biofilm. Front Microbiol..

[27] Candela T., Fagerlund A., Buisson C., Gilois N., Kolsto A.B., Okstad O.A., Aymerich S., Nielsen-Leroux C., Lereclus D., Gohar M. (2019). CalY is a major virulence factor and a biofilm matrix protein. Mol. Microbiol..

[28] Fagerlund A., Dubois T., Okstad O.A., Verplaetse E., Gilois N., Bennaceur I., Perchat S., Gominet M., Aymerich S., Kolsto A.B. (2014). SinR controls enterotoxin expression in *Bacillus thuringiensis* biofilms. PLoS ONE.

[29] Auger S., Ramarao N., Faille C., Fouet A., Aymerich S., Gohar M. (2009). Biofilm formation and cell surface properties among pathogenic and nonpathogenic strains of the *Bacillus cereus* group. Appl. Environ. Microbiol..

[30] Gohar M., Gilois N., Graveline R., Garreau C., Sanchis V., Lereclus D. (2005). A comparative study of *Bacillus cereus*, *Bacillus thuringiensis* and *Bacillus anthracis* extracellular proteomes. Proteomics.

[31] Koza A., Moshynets O., Otten W., Spiers A.J. (2011). Environmental modification and niche construction: Developing O_2_ gradients drive the evolution of the Wrinkly Spreader. ISME J..

[32] Duport C., Thomassin S., Bourel G., Schmitt P. (2004). Anaerobiosis and low specific growth rates enhance hemolysin BL production by Bacillus cereus F4430/73. Arch. Microbiol..

[33] Mampel J., Spirig T., Weber S.S., Haagensen J.A., Molin S., Hilbi H. (2006). Planktonic replication is essential for biofilm formation by *Legionella pneumophila* in a complex medium under static and dynamic flow conditions. Appl. Environ. Microbiol..

[34] Habimana O., Meyrand M., Meylheuc T., Kulakauskas S., Briandet R. (2009). Genetic features of resident biofilms determine attachment of *Listeria Monocytogenes*. Appl. Environ. Microbiol..

[35] Houry A., Gohar M., Deschamps J., Tischenko E., Aymerich S., Gruss A., Briandet R. (2012). Bacterial swimmers that infiltrate and take over the biofilm matrix. Proc. Natl. Acad. Sci. USA.

[36] Giglio K.M., Fong J.C., Yildiz F.H., Sondermann H. (2013). Structural basis for biofilm formation via the *Vibrio cholerae* matrix protein RbmA. J. Bacteriol..

[37] Smith D.R., Maestre-Reyna M., Lee G., Gerard H., Wang A.H., Watnick P.I. (2015). *In situ* proteolysis of the *Vibrio cholerae* matrix protein RbmA promotes biofilm recruitment. Proc. Natl. Acad. Sci. USA.

[38] Wijman J.G., de Leeuw P.P., Moezelaar R., Zwietering M.H., Abee T. (2007). Air-liquid interface biofilms of *Bacillus cereus*: Formation, sporulation, and dispersion. Appl. Environ. Microbiol..

[39] Vlamakis H., Chai Y., Beauregard P., Losick R., Kolter R. (2013). Sticking together: Building a biofilm the *Bacillus subtilis* way. Nat. Rev. Microbiol..

