# Supplementary material for: Massive Integration of Planktonic Cells within a Developing Biofilm
Source: Microorganisms. 2021 Feb 2;9(2):298. doi: 10.3390/microorganisms9020298 (PMC7912878; doi:10.3390/microorganisms9020298)
Supplement: Supplementary file 1 [file microorganisms-09-00298-s001.zip › Table S2.docx]

| Primer names | Primer sequences | Restriction sites |
| --- | --- | --- |
| AMYAFW | 5'-**CGGGATCC**CAACTCCGGAAGTGATGTGAA-3' | BamHI |
| AMYARV | 5'-**TTGGCGCGCC**CGCACGTCTACTCCATCG-3' | AscI |
|  |  |  |
| AMYBFW | 5'-**GGTAAC**AACGAACAACAGGTAATTGATGCAAT-3' | BstEII |
| AMYBRV | 5'-**CCAAGCTT**CATTCACACCAACGATTAAGGAGAT-3' | HinDIII |
|  |  |  |
| SGFPFW | 5'-**TTGGCGCGC**CGCCAGTGCCAAGCTTCTG-3' | AscI |
| SGFPRV | 5'-**GGGTAAC**CGCTTCCGGCTCGTATGTTGTG-3' | BstEII |
|  |  |  |
| pAphaIII-F | 5’-acat**gcatgc**GAACCATTTGAGGTGATAGG-3’ | SphI |
| pAphaIII-R | 5’-**TCTAGA**CTTTTCTACAGTATTT-3’ | XbaI |
|  |  |  |
| mCherry-F | 5’-**GGTACC**ACATAAGGAGGAACTA-3’ | KpnI |
| mCherry4 | 5’-cg**GAATTC**TTACTTATATAATTC-3’ | EcoRI |

Table S2 : Primers used in this study. Bold letters represent the restriction sites
