# Supplementary material for: Massive Integration of Planktonic Cells within a Developing Biofilm
Source: Microorganisms. 2021 Feb 2;9(2):298. doi: 10.3390/microorganisms9020298 (PMC7912878; doi:10.3390/microorganisms9020298)
Supplement: Supplementary file 1 [file microorganisms-09-00298-s001.zip › Table S1.docx]

| Strain | species | description | reference |
| --- | --- | --- | --- |
| *Bt407* | *B. thuringiensis* | wild-type strain | 15 |
| *Bt407 ∆Spo0A* | *B. thuringiensis* | Insertion of a kanamycin resistance cassette in *spo0A* | 17 |
| *Bt407-sGfp* | *B. thuringiensis* | Constitutive expression of GFP | this study |
| *Bt407-mCherry* | *B. thuringiensis* | Constitutive expression of mCherry | this study |
| ATCC14579 | *B. cereus* | wild-type strain | ATCC collection |
| ATCC10987 | *B. cereus* | wild-type strain | ATCC collection |
| AH829 | *B. cereus* | wild-type strain | 18 |

Table S1 : Strains used in this study
