# Supplementary material for: Massive Integration of Planktonic Cells within a Developing Biofilm
Source: Microorganisms. 2021 Feb 2;9(2):298. doi: 10.3390/microorganisms9020298 (PMC7912878; doi:10.3390/microorganisms9020298)
Supplement: Supplementary file 1 [file microorganisms-09-00298-s001.zip › Figure S1.pdf]

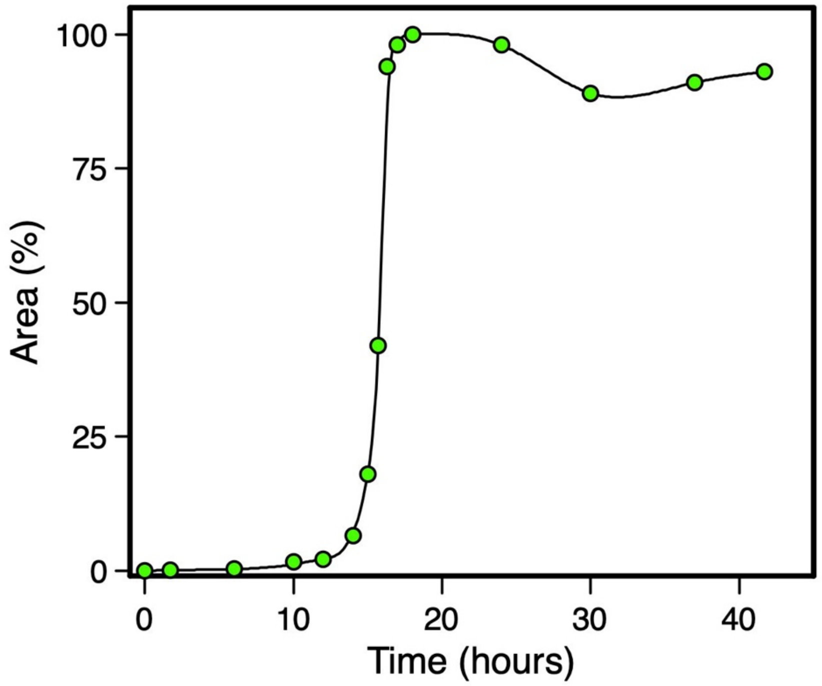

<u>Figure S1</u> : Area covered by the biofilm as a function of time.
Images taken from video S3 were converted to black and white pictures, substracted with the time 0 image, and analyzed by ImageJ to determine the number of pixels which display a value above the empty surface background (pixel values from 20 to 255, 0 is black and 255 is white). The number of pixels obtained was then converted to the percentage of surface occupied by the biofilm (Area %, Y-axis)
