# Supplementary material for: Massive Integration of Planktonic Cells within a Developing Biofilm
Source: Microorganisms. 2021 Feb 2;9(2):298. doi: 10.3390/microorganisms9020298 (PMC7912878; doi:10.3390/microorganisms9020298)
Supplement: Supplementary file 1 [file microorganisms-09-00298-s001.zip › Figure S2.pdf]

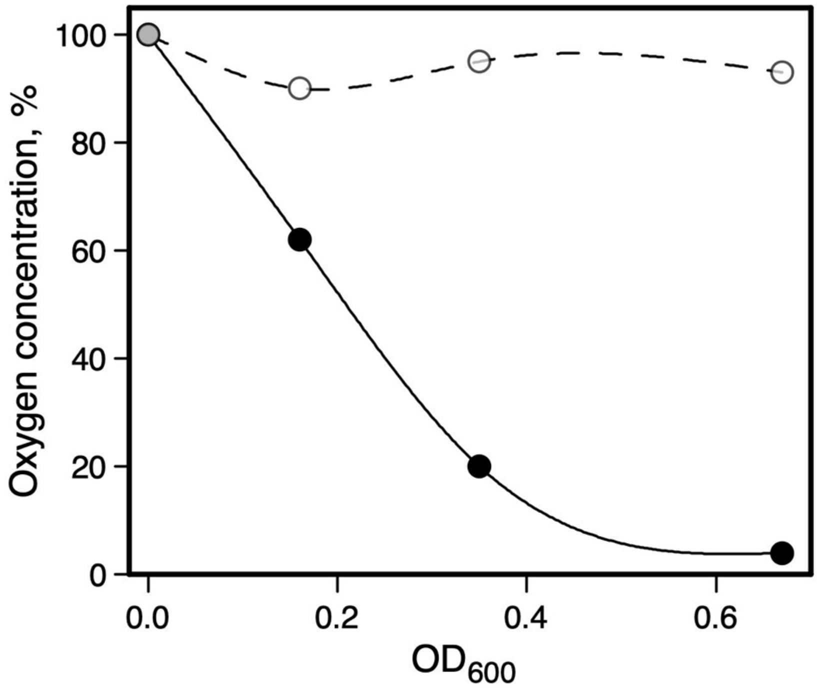

Figure S2 : Dissolved oxygen concentration in the culture medium during planktonic growth. A 25 ml planktonic culture of the *B. thuringiensis* 407 strain, seeded at OD_{600} 0.01 in HCT medium, was grown at 30°C in static conditions in 2.5 cm diameter polyethylene tubes. No biofilm could be produced in these conditions. The dissolved oxygen was measured at the tube mid-height with a Mettler-Toledo oxymeter Seven2Go equipped with an Inlab 605-ISM electrode, at different times. One tube was used per measurement. Black circles : dissolved oxygen in the planktonic culture. Open circles : unseeded tubes, used as controls. The dissolved oxygen concentration at time zero was set to 100%. The oxygen concentration is expressed as a function of the OD_{600} of the planktonic population at the time of measurement.
